# Safety, pharmacokinetics and pharmacodynamics of single rising doses of BI 655064, an antagonistic anti-CD40 antibody in healthy subjects: a potential novel treatment for autoimmune diseases

**DOI:** 10.1007/s00228-017-2362-8

**Published:** 2017-11-10

**Authors:** Fredrik N. Albach, Frank Wagner, Andreas Hüser, Julia Igel, David Joseph, James Hilbert, Corinna Schoelch, Steven J. Padula, Jürgen Steffgen

**Affiliations:** 1 Charité Research Organisation GmbH, Berlin, Germany; 2 Boehringer Ingelheim Pharma GmbH & Co. KG, Birkendorfer Str. 65, Biberach/Riss, Germany; 3 Boehringer Ingelheim Pharmaceuticals, Inc., Ridgefield, CT USA; 4 Boehringer Ingelheim Pharma GmbH & Co. KG, Ingelheim, Germany

**Keywords:** Anti-CD40, Monoclonal antibody, First-in-human trial, Rheumatoid arthritis, Systemic lupus erythematosus, Lupus nephritis

## Abstract

**Purpose:**

The CD40–CD40L pathway is a promising treatment target for autoimmune diseases such as rheumatoid arthritis, systemic lupus erythematosus and lupus nephritis. The safety, pharmacokinetics and pharmacodynamics of BI 655064, a novel humanised antagonistic anti-CD40 monoclonal antibody, were investigated in this first-in-human trial.

**Methods:**

Healthy male subjects (*n* = 72) were randomised 3:1, within each BI 655064 dose group, to single intravenous (IV; 0.2–120 mg) or subcutaneous (SC; 40–120 mg) doses of BI 655064 or placebo. Safety, plasma exposure, CD40 receptor occupancy and CD40L-induced CD54 upregulation were assessed over 12 weeks.

**Results:**

Adverse events (AEs) were reported in 43% of subjects (*n* = 31). Frequency and intensity of AEs were generally similar between BI 655064 and placebo and showed no dose relationship. The most frequent AEs were headache and nasopharyngitis. One mild rash and one local reaction occurred with SC BI 655064; two serious AEs were reported, both judged unrelated to BI 655064. Pharmacokinetic evaluation demonstrated a more than proportional increase in plasma exposure relative to BI 655064 dose, with a terminal half-life between 4 h and 4 days IV and approximately 5 days SC; doses ≥ 20 mg IV and 120 mg SC showed > 90% CD40 receptor occupancy and inhibition of CD54 upregulation, which lasted 7 days in the 120 mg IV and SC groups.

**Conclusions:**

Single doses up to 120 mg BI 655064 IV and SC were well tolerated and showed a high potential to block the CD40–CD40L pathway, supporting further clinical development of BI 655064 in patients with autoimmune disease.

**Trial registration:**

ClinicalTrials.gov Identifier: NCT01510782

**Electronic supplementary material:**

The online version of this article (10.1007/s00228-017-2362-8) contains supplementary material, which is available to authorized users.

## Introduction

Despite therapeutic progress in recent years, there is still a need for new treatments for autoimmune diseases such as rheumatoid arthritis (RA) and, in particular, systemic lupus erythematosus (SLE) and lupus nephritis (LN) [1, 2]. The interaction of the cell surface receptor CD40 and its ligand CD40L (CD154) is known to play a central role in the regulation of humoral and cellular immunity and in the pathogenesis of these autoimmune diseases [3, 4]; thus, targeting this interaction may be a therapeutic option.

CD40 is a 48-kDa type I membrane glycoprotein of the tumour necrosis factor (TNF) receptor superfamily expressed on B cells, dendritic cells (DCs), monocytes, macrophages and other non-immune cells [3, 5]. CD40, a key co-stimulatory molecule involved in the development of antigen-driven acquired immunity by activating B cells and other antigen-presenting cells, including DCs and macrophages, is also involved in the activation of non-immune resident cells in the kidney [4, 6]. CD40L is a TNF superfamily member expressed on activated CD4+ T cells, a small subset of CD8+ T cells and activated platelets [4, 5].

The generation of autoantibodies, characteristic of RA, SLE and LN, is dependent on T cell–B cell interactions within the lymph node and requires CD40–CD40L engagement to drive B cell proliferation and formation of germinal centres [7]. CD40–CD40L interaction also causes upregulation of intercellular adhesion molecules, such as CD54 on immune cells, endothelial cells and synovial fibroblasts [8, 9]. Increased expression of CD40L on T cells has been shown to correlate with higher disease activity in patients with RA [10]. Gene analyses have demonstrated an association of CD40 polymorphisms with susceptibility to RA [11], the presence of rheumatoid factor, anti-cyclic citrullinated peptide antibodies [12] and a higher rate of joint destruction in patients with RA [13]. In patients with SLE, increased B cell expression of CD40L results in spontaneous autoantibody production in a T cell-independent manner [7]. In SLE and LN, CD40 and CD40L expression is elevated [7, 14], and increased CD40L expression on monocytes is associated with higher disease activity [15]. Furthermore, human kidney mesangial cells express low levels of CD40 and upregulate this receptor in response to interferon-γ treatment and activated CD40L+ platelets from patients with SLE [6].

Previous clinical development of monoclonal antibodies against CD40L (BG9588 and IDEC-131) in SLE has been complicated by cardiovascular thromboembolic events, which may have been caused by immune complexes consisting of soluble CD40L and anti-CD40L antibodies [16–18]. It was later clarified that wild-type Fc-mediated signalling, through FcR II, fully activates platelets and induces aggregation [19, 20] and that reducing FcR binding eliminates the ability for platelet activation [21, 22]. An alternative approach to block CD40–CD40L interaction is to target CD40. This approach has not been associated with thromboembolism in recent clinical trials. The therapeutic use of antagonistic anti-CD40 antibodies has been investigated in phase 1 trials in healthy subjects [23], in patients with Crohn’s disease [24] and in cancer patients [25].

BI 655064 is a humanised antagonistic monoclonal antibody (IgG1) that selectively binds CD40 and blocks CD40–CD40L interaction. BI 655064 was designed to have no agonistic activity and to prevent stimulating cytokine production; two replacement mutations in the Fc region (Leu234Ala and Leu235Ala) were also incorporated to prevent Fc-mediated cytotoxicity and platelet activation. BI 655064 demonstrated potent and comparable binding properties in both human and cynomolgus monkey B cells (EC90 = 6.85 ± 0.74 nM) and potent inhibition of CD40L-induced peripheral blood mononuclear cell proliferation without agonism. When bound to platelets, BI 655064 does not appear to alter platelet activation, aggregation or function [26]. In pre-clinical assessments in cynomolgus monkeys, with multiple doses up to 26 weeks and up to 50 mg/kg, reversible decreases in B cell levels, reversible reduction of lymphoid organ germinal centres and good general tolerability without thromboembolic events or relevant cytokine release were demonstrated ([26] and unpublished data). The no observed adverse effect level in these assessments was 50 mg/kg (unpublished data). Here, the findings of a first-in-human phase 1 trial of single rising intravenous (IV) or subcutaneous (SC) BI 655064 doses in healthy subjects are reported. The primary objective was to investigate the safety and tolerability of BI 655064, and secondary objectives included exploring the pharmacokinetics (PK) and pharmacodynamics (PD), CD40 receptor occupancy and inhibition of CD54 upregulation after IV and SC doses of BI 655064.

## Methods

### Study participants

In total, 72 healthy male subjects aged 18–55 years with body mass indices (BMI) between 18.5 and 29.9 kg/m^2^ were enrolled in the study. The main exclusion criteria were clinically relevant pre-existing diseases or abnormalities in the screening examination. These included urine drug tests and infectious serology (hepatitis, HIV, tuberculosis), confirmed prolongation of QT/QTc (e.g. repeated demonstration of a QTc interval > 450 ms), increased risk of bleeding (assessed by medical history, family history, deficiency of antithrombin III or protein S or C) and prolonged bleeding time ≥ 6 min.

### Study design

This clinical phase 1 study was a randomised, single-blind, placebo-controlled trial with single rising doses of BI 655064. The primary objective was to investigate the safety and tolerability after single doses of BI 655064; secondary objectives were to explore the PK, PD and bioavailability after SC injections. Subject eligibility was determined at screening (up to 21 days before randomisation) and confirmed at baseline before the first dose. Eligible subjects were randomised to receive BI 655064 or placebo in seven IV dose groups and three SC dose groups. The first two dose groups (0.2 and 0.6 mg IV) comprised four subjects (three active, one placebo), whereas all other dose groups comprised eight subjects (six active, two placebo). The dose levels were 0.2, 0.6, 2, 6, 20, 60 and 120 mg for the IV groups and 40, 80 and 120 mg for the SC groups. Based on results from dosing in cynomolgus monkeys, the IV starting dose of 0.2 mg was selected because this was the lowest dose that could lead to quantifiable serum concentrations and was expected to result in 20% receptor occupancy, thus having minimal biologic effects. This starting dose is 15,152-fold below the no observed adverse effect level in the cynomolgus monkey (50 mg/kg) and offered a broad safety margin while providing meaningful PK and PD data. For SC, the starting dose of 40 mg was based on human PK modelling, which predicted that an SC dose of 50 mg once weekly would be required to maintain sufficient receptor occupancy of over 90%. There was a minimum observation period of at least 22 h between each of the first four subjects in each dose group. Escalation to the next dose level was decided by an independent drug safety monitoring board, based on evaluation of safety, tolerability and laboratory parameters. For the IV application, 10 mg/ml BI 655064 was diluted with NaCl 0.9% and given as a 40 ml infusion over 60 min, whereas the SC application was given at a concentration of 120 mg/ml in the abdominal subcutis. Subjects were hospitalised for 48 h (IV groups) or 72 h (SC groups) after drug administration. They then had nine ambulatory follow-up visits over a total observation period of 70 days after study drug administration, unless adverse events (AEs) warranted further observation.

Blood samples (2.7 ml) for PK analysis were collected from a forearm vein using an indwelling catheter into tripotassium ethylenediaminetetraacetic acid anticoagulant tubes. For IV administration, the time points for sample collection were pre-infusion (− 15 min), during the infusion (30 min and 1 h) and at the following time points post-infusion 1.25, 1.5, 1.75, 2, 2.5, 3, 4, 6, 8, 10 and 12 h and 1, 1.25, 1.5, 2, 4, 6, 8, 12, 19, 28, 42 and 56 days. For the early time points after drug administration, PK samples were to be obtained from the forearm not used in the infusion. For SC administration, the time points for sample collection were pre-injection (− 15 min) and at the following time points post-injection 0.5, 1, 1.5, 2, 3, 4, 6, 8, 10 and 12 h and 1, 1.5, 2, 3, 4, 5, 7, 11, 18, 27, 41 and 55 days. The study was sponsored by Boehringer Ingelheim and conducted at a single trial centre in Berlin, Germany, by Charité Research Organisation GmbH. This study was approved by the local ethics committee and the German health authority, and all subjects provided written informed consent. The study registration identifier is ClinicalTrials.gov NCT01510782.

### Study assessments

Safety and tolerability measures included the assessment of AEs, physical examination, vital signs (blood pressure and pulse), 12-lead electrocardiograms (ECGs), laboratory tests and cytokine measurements (biochemistry, haematology, urinalysis, interleukins (IL)-2, -4, -6, -8, -10 and -12, TNFα, interferon-γ, complement factors C3 and C5a, adhesion molecules E-/P-selectin and B cell subsets). To examine the potential for thromboembolic events, the following evaluations were performed: international normalised ratio (INR), activated partial thromboplastin time (aPTT), antithrombin III, protein S and C, percentage of platelet aggregation induced by adenosine diphosphate (ADP), time to platelet aggregation measured with a platelet function analyser (PFA100), bleeding time (measured with the Duke method) and D-dimers. Serum anti-drug (BI 655064) antibodies (ADAs) were examined using a validated bridging electrochemiluminescence assay (in-house assay; Boehringer Ingelheim, Biberach, Germany) on study days 1, 7 and 70 (days 1 and 7 in the 40 mg SC group). A sample was considered ADA positive if its response in the screening assay was greater than or equal to a plate-specific cut point and if it was confirmed positive in a specificity test (response blocked by addition of BI 655064). Confirmed ADA-positive samples were further characterised in a titre assay. Titres were determined by analysis of serial twofold dilutions of a sample. The reported titre was the highest fold-dilution that produced a mean electrochemiluminescent value greater than or equal to the confirmatory cut point. Plasma concentrations of BI 655064 were assessed at all visits using a validated sandwich enzyme-linked immunosorbent assay (in-house assay, Boehringer Ingelheim, Biberach, Germany) with a lower limit of quantification of 30 ng/ml. The 96-well microtiter plates were first coated with an anti-BI 655064 antibody (Boehringer Ingelheim, Biberach, Germany), blocked and washed. The plates were then incubated with study samples, calibrators or quality control samples and then washed again. Binding of BI 655064 was detected with a biotinylated anti-BI 655064 antibody (Boehringer Ingelheim, Biberach, Germany) followed by streptavidin conjugated with horseradish peroxidase and finally with the peroxidase substrate tetramethylbenzidine. Plates were read colourimetrically, with data analysed with a 5-parameter logistic fit. The quantitative range was 30–800 ng/ml. Maximum plasma concentration (*C*_max_) and time to *C*_max_ (*t*_max_) were the observed values. Further PK parameters were calculated according to standard non-compartmental methods and included area under the plasma concentration–time curve (AUC), terminal half-life (*t*_1/2_), drug clearance (CL or CL/F for SC subjects) and volume of distribution during the terminal phase (*V*_z_ or *V*_z_/*F* for SC subjects). Both the determination of BI 655064 concentrations and the ADA assessments were performed by Covance Laboratories, Inc. (Chantilly, VA, USA).

After dosing, PD was analysed for up to 1 week in IV cohorts and up to 11 days in SC cohorts. For measurement of CD40 receptor occupancy, whole blood samples were incubated with an excess of fluorescein isothiocyanate (FITC)-labelled BI 655064 (Boehringer Ingelheim, Biberach, Germany) and anti-CD19 APC (BD, Heidelberg, Germany) for B cell gating. The fluorescent signals on B cells are directly proportional to the number of unbound CD40 receptors and allow an indirect assessment of CD40 receptor occupancy by BI 655064. To assess CD40–CD40L interaction, whole blood samples were incubated with IL-4 (ProSpec, Rehovot, Israel) and MegaCD40L (Alexis Biochemicals, Lörrach, Germany) for 24 h at 37 °C to induce CD54 upregulation and then stained with anti-CD19 APC and anti-CD54 PE (BD, Heidelberg, Germany) for measurement of CD54 expression. For both PD assays, the fluorescent signals on B cells measured by flow cytometry at each time point were compared with pre-dose samples to determine the percentage of change.

The relationships between the dose of BI 655064 and inhibition of CD40 receptor occupancy and CD54 upregulation were explored using standard sigmoidal *E*_max_ models, where *E*_max_ is the maximum effect at *C* = infinity, EC_50_ is the estimated half-maximal effective concentration and gamma is the sigmoidicity (shape) parameter.

### Statistical analysis

Study results were analysed using descriptive statistics for safety, PK and PD. The safety population included all subjects who had received the study drug. The PK and PD populations included all subjects who had received the study drug and who provided evaluable data for PK and PD analysis without important protocol violations relevant for PK and PD. The dose proportionality was assessed using a power model, whereas absolute bioavailability was determined using an analysis of variance model.

## Results

### Study participants

In total, 163 subjects were screened and 72 healthy subjects were randomised and enrolled in the trial, including 48 subjects who received IV treatment with placebo (*n* = 12) or BI 655064 (*n* = 36) and 24 subjects who received SC treatment with placebo (*n* = 6) or BI 655064 (*n* = 18). Two subjects withdrew consent after receiving the trial medication; 70 subjects completed the trial as planned. All 72 treated subjects were included in the safety and PD analyses and all 54 subjects with active treatment were included in the PK analyses. All subjects were male with a mean (standard deviation) age of 39.1 (8.4) years and a mean (standard deviation) BMI of 25.2 (2.7) kg/m^2^ (online resource 1 Supplementary Table S1). Characteristics did not show major differences between groups. All subjects were Caucasian except one subject of African descent in the placebo IV dose group.

### Safety and tolerability

Overall, 43 AEs were reported in 31 subjects after treatment (43% of subjects). The proportion of subjects experiencing AEs was generally similar after BI 655064 administration and after placebo (22 active subjects [41%] vs 9 placebo subjects [50%]) and showed no relationship to dose. The most frequent AEs were headache, nasopharyngitis and oropharyngeal pain/sore throat (Table 1). Headache was more commonly reported in subjects treated with BI 655064 (nine active subjects [17%] vs one placebo subject [6%]) whereas the incidence of nasopharyngitis was similarly distributed between the groups (five active subjects [9%] vs two placebo subjects [11%]). There were three reports of oropharyngeal pain in the active group (6%). Two subjects treated with 40 mg BI 655064 SC had drug-related AEs. These were a mild rash that lasted for 1 month and a mild local injection site erythema after SC injection, which lasted for 2 days. Serious AEs (SAEs) requiring hospitalisation were reported for one placebo and one active subject (3%). The subject on active treatment (60 mg BI 655064 IV) had a rupture of the deltoid ligament of the left foot and subsequent oedema due to a pronation trauma, which was initially diagnosed as skin infection and treated with antibiotics. This event was not judged to be related to the trial medication. Apart from the SAEs, all AEs were of mild or moderate severity. There were no deaths during the study and no subjects discontinued because of an AE.

There were no clinically relevant findings or treatment differences between groups with regard to vital signs, ECG or the physical examination. Local tolerability revealed local pain at the site of IV infusion in two subjects (before infusion in one subject) and a local erythema at the site of SC injection in one subject. There were no clinically relevant changes in the safety laboratory evaluations. D-dimer values varied and increased to above normal range in four active (7%) and four placebo subjects (22%) after treatment (all < 1.5 × upper limit of normal). A few subjects in each of the placebo and the BI 655064 groups had mild decreases of time to platelet aggregation but values remained within the normal range. None of these changes were seen as clinically significant or reported as AEs. There were no relevant changes in aPTT, percentage of ADP-induced platelet aggregation or bleeding time and no clinical signs of thromboembolic events or bleeding in any subject. Platelet counts over time remained stable across all IV and SC doses. Sub-analysis of platelet counts by ADA status revealed no ADA-dependent impact on total platelet count over time. No significant changes or trends were observed in cytokines, complement factors or adhesion molecules, lymphocytes or B cell phenotypes (naive [CD19+ CD27−], memory [CD19+ CD27+] and plasmablasts/plasma cells [CD19+ CD27^bright^ CD38^bright^]), and there was no clinical evidence of cytokine release or hypersensitivity.

ADA levels remained negative or unchanged in all subjects receiving placebo at all time points. Pre-existing ADAs were found in six subjects (8%). Seroconversion within the first week after dosing with BI 655064 occurred in one subject (dose level 0.2 mg IV, titre = 1). Until study day 70, treatment-induced or treatment-boosted ADAs were detected in 13 (36%) subjects dosed with IV BI 655064 and in 6 (50%) subjects dosed with SC BI 655064. The highest rate of seroconversion was seen in the 120 mg IV group (five subjects, 83%) and the highest titres observed were 200 in one subject in each of the 120 mg IV and 120 mg SC group and 400 in the 80 mg SC group.

### Pharmacokinetics

**Fig. 1:**
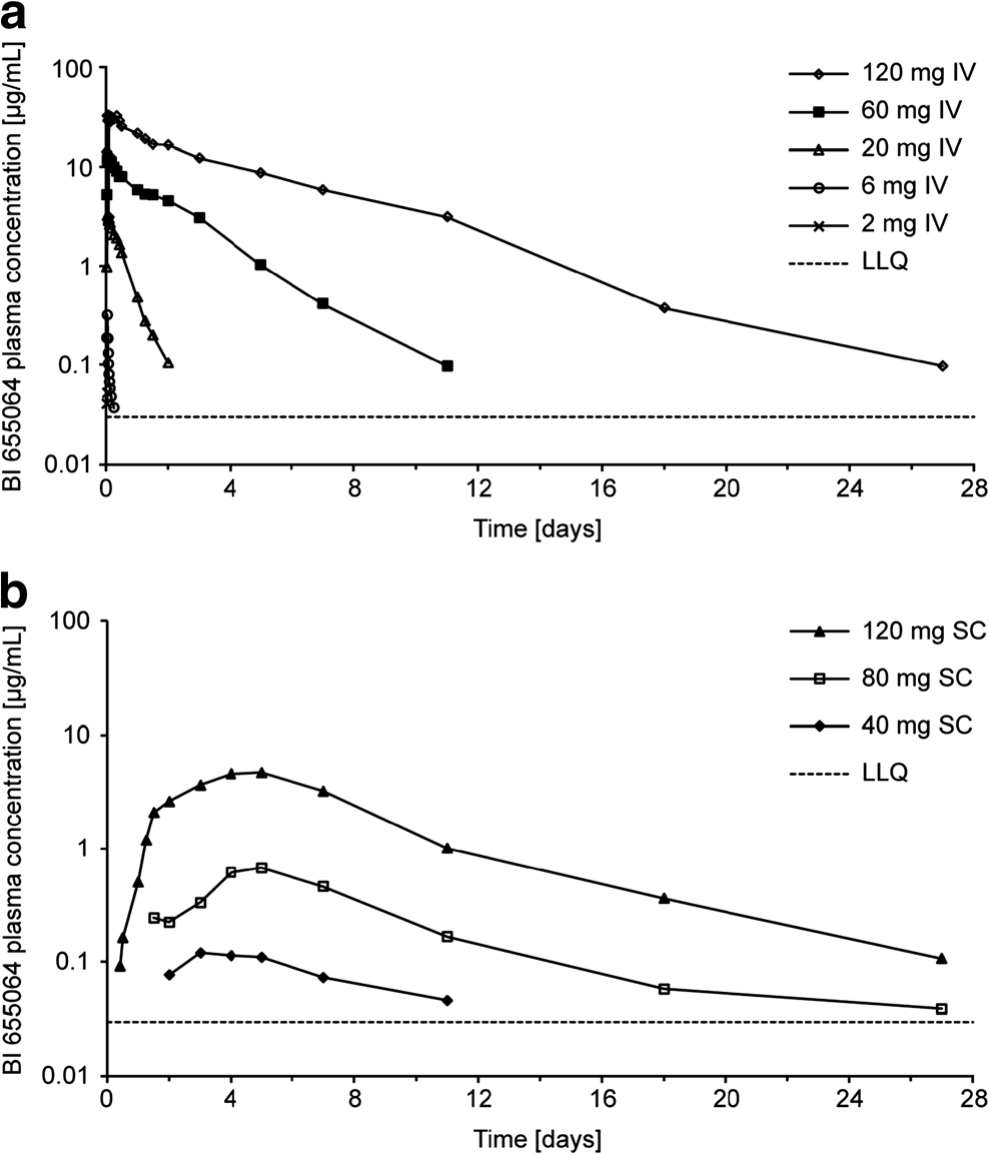
Semi-log plots for geometric BI 655064 mean plasma concentrations after IV (**a**) or SC (**b**) administration. Arithmetic mean (SD) plasma concentrations of BI 655064 are provided in online resource 1 Supplemental Tables S3 and S4. *IV* intravenous, *LLQ* lower limit of quantification, *SC* subcutaneous

After IV infusion, BI 655064 concentrations were not measurable in the 0.2 and 0.6 mg dose groups and in all but a few isolated samples of the 2 mg dose group. Peak plasma concentrations were reached at the end of infusion (*t*_max_ = 1 h) in the 2 and 6 mg dose groups and up to 3 h after the start of infusion in the 60 and 120 mg dose groups. Concentrations decreased rapidly thereafter. Increase in plasma exposure was substantially more than proportional to dose. With a 20-fold increase in dose from 6 mg to 120 mg, the geometric mean (gmean) *C*_max_ increased from 318 to 35,800 ng/ml. Over the dose range 2–120 mg, the slopes (*β*) for *C*_max_ and AUC_0−∞_ vs dose plots (1.6 [95% confidence interval (CI) 1.5–1.7] and 2.8 [95% CI 2.6–2.9], respectively) were well above unity. Clearance showed a dose-dependent decrease from 139 ml/min at the 6 mg dose to 0.672 ml/min at the 120 mg dose, and the gmean *t*_½_ increased in a dose-related fashion from 4 h to 4 days (Table 2 and Fig. 1). After SC injection, BI 655064 was absorbed slowly from the site of injection (median *t*_max_ 4–5 days). The gmean *t*_½_ was 5–6 days, and the slope *β* was 3.3 for *C*_max_ (95% CI 2.2–4.4) and 3.1 for AUC_0−∞_ (95% CI 1.8–4.5). The bioavailability of the 120 mg SC dose, compared with the 120 mg IV dose, was 13.9% for *C*_max_ (90% CI 7.5–25.7) and 30.3% for AUC_0−∞_ (90% CI 13.7–66.9). PK variability was higher after SC dosing than after IV dosing.

**Table 1:** Summary of adverse events

*n* (%)	Placebo		BI 655064										
			IV							SC			IV + SC (*n* = 54)
	IV (*n* = 12)	SC (*n* = 6)	0.2 mg (*n* = 3)	0.6 mg (*n* = 3)	2 mg (*n* = 6)	6 mg (*n* = 6)	20 mg (*n* = 6)	60 mg (*n* = 6)	120 mg (*n* = 6)	40 mg (*n* = 6)	80 mg (*n* = 6)	120 mg (*n* = 6)	
Any AEs	6 (50)	3 (50)	1 (33)	2 (67)	2 (33)	3 (50)	0	3 (50)	4 (67)	3 (50)	1 (17)	3 (50)	22 (41)
Severe AEs	1 (8)	0	0	0	0	0	0	1 (17)	0	0	0	0	1 (2)
Serious AEs^a^	1 (8)	0	0	0	0	0	0	1 (17)	0	0	0	0	1 (2)
Most common AEs^b^													
Headache	0	1 (17)	1 (33)	1 (33)	1 (17)	1 (17)	0	1 (17)	0	1 (17)	1 (17)	2 (33)	9 (17)
Nasopharyngitis	1 (8)	1 (17)	0	0	1 (17)	0	0	0	2 (33)	1 (17)	0	1 (17)	5 (9)
Oropharyngeal pain	0	0	0	1 (33)	0	0	0	1 (17)	0	1 (17)	0	0	3 (6)

^a^Both subjects experiencing a serious AE required hospitalisation

^b^AEs occurring in ≥ 2 subject receiving BI 655064 are reported

**Table 2:** Pharmacokinetic parameters

	*C*_max_ [ng/ml]	AUC_0−tz_ [ng x h/ml]	AUC_0−∞_ [ng x h/ml]	*t*_max_^a^ [h]	*t*_1/2_ [h]	CL^b^ [ml/min]	*V*_z_^b^ [L]
2 mg IV (*n* = 6)							
gmean	51.5	32.9	NC	1.00	NC	NC	NC
gCV (%)	16.9	19.3	NC	1.00–1.00	NC	NC	NC
6 mg IV (*n* = 6)							
gmean	318	507	721	1.00	3.93	139	47.2
gCV (%)	18.3	34.4	22.4	1.00–1.02	26.6	22.4	34.9
20 mg IV (*n* = 6)							
gmean	3410	43,100	44,400	1.38	15.6	7.50	10.1
gCV (%)	36.7	74.1	71.6	1.00–2.00	25.1	71.6	90.1
60 mg IV (*n* = 6)							
gmean	12,900	586,000	590,000	1.63	45.6	1.69	6.69
gCV (%)	18.4	38.7	38.5	1.00–3.17	23.6	38.5	45.4
120 mg IV (*n* = 6)							
gmean	35,800	2970,000	2,980,000	1.25	87.4	0.672	5.08
gCV (%)	14.4	20.0	20.0	1.00–3.00	22.7	20.0	15.5
40 mg SC (*n* = 5)							
gmean	122	18,000	NC	96.1	NC	NC	NC
gCV (%)	94.1	73.2	NC	72–168	NC	NC	NC
80 mg SC (*n* = 6)							
gmean	717	120,000	130,000	120	138	10.2	122
gCV (%)	151	134	119	96.1–120	25.1	119	150
120 mg SC (*n* = 6)							
gmean	4970	888,000	900,000	108	117	2.22	22.6
gCV (%)	97.9	144	143	95.8–120	101	143	83.4

Arithmetic means (SDs) for pharmacokinetic parameters are provided in online resource 1 Supplemental Table S2

*AUC*_*0−∞*_ area under the concentration–time curve of the analyte in plasma over the time interval from 0 to extrapolated to infinity, *AUC*_*0−tz*_ area under the concentration–time curve of the analyte in plasma over the time interval from 0 to the last measurable time point of the dose, *CL* total clearance of the analyte in plasma after intravascular administration, *C*_*max*_ maximum measured concentration of the analyte in plasma, *gCV* geometric coefficient of variation, *gmean* geometric mean, *IV* intravenous, *NC* not calculated, *SC* subcutaneous*, t*_*1/2*_ terminal elimination half-life of the analyte in plasma, *t*_*max*_ time from dosing to the maximum measured concentration of the analyte in plasma or the maximum measured biomarker effect, *V*_*z*_ apparent volume of distribution during the terminal phase after an intravascular dose

^a^Data for *t*_max_ are presented as median (range)

^b^CL and *V*_z_ expressed as functions of bioavailability (CL/*F* and *V*_z_/*F*) for SC parameters

### Pharmacodynamics

**Fig. 2:**
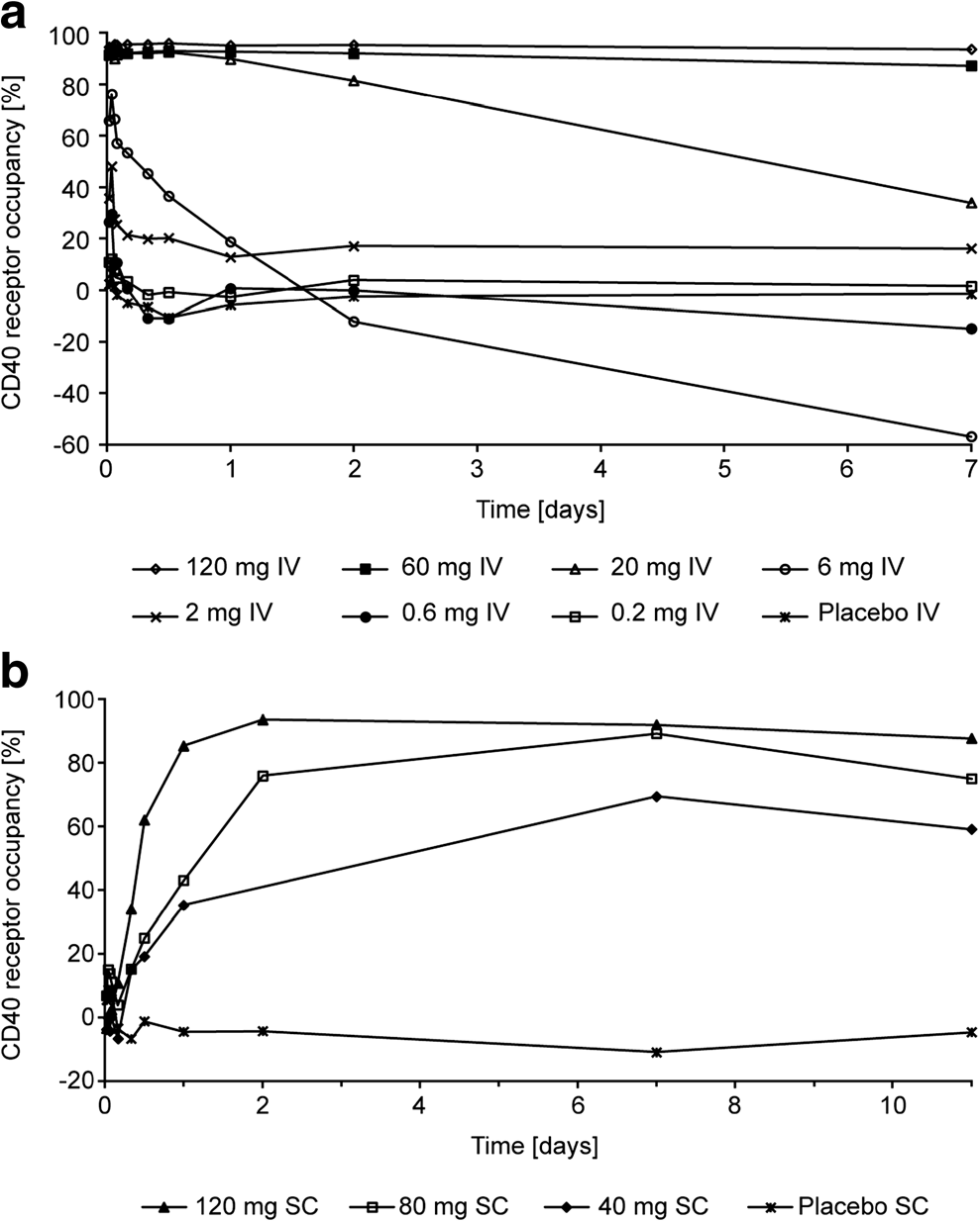
Arithmetic mean percentage of CD40 receptor occupancy over time after IV (**a**) or SC (**b**) administration of BI 655064. Tabulated values (mean and SD) are provided in online resource 1 Supplemental Tables S5 and S6. *IV* intravenous, *SC* subcutaneous, *SD* standard deviation

**Fig. 3:**
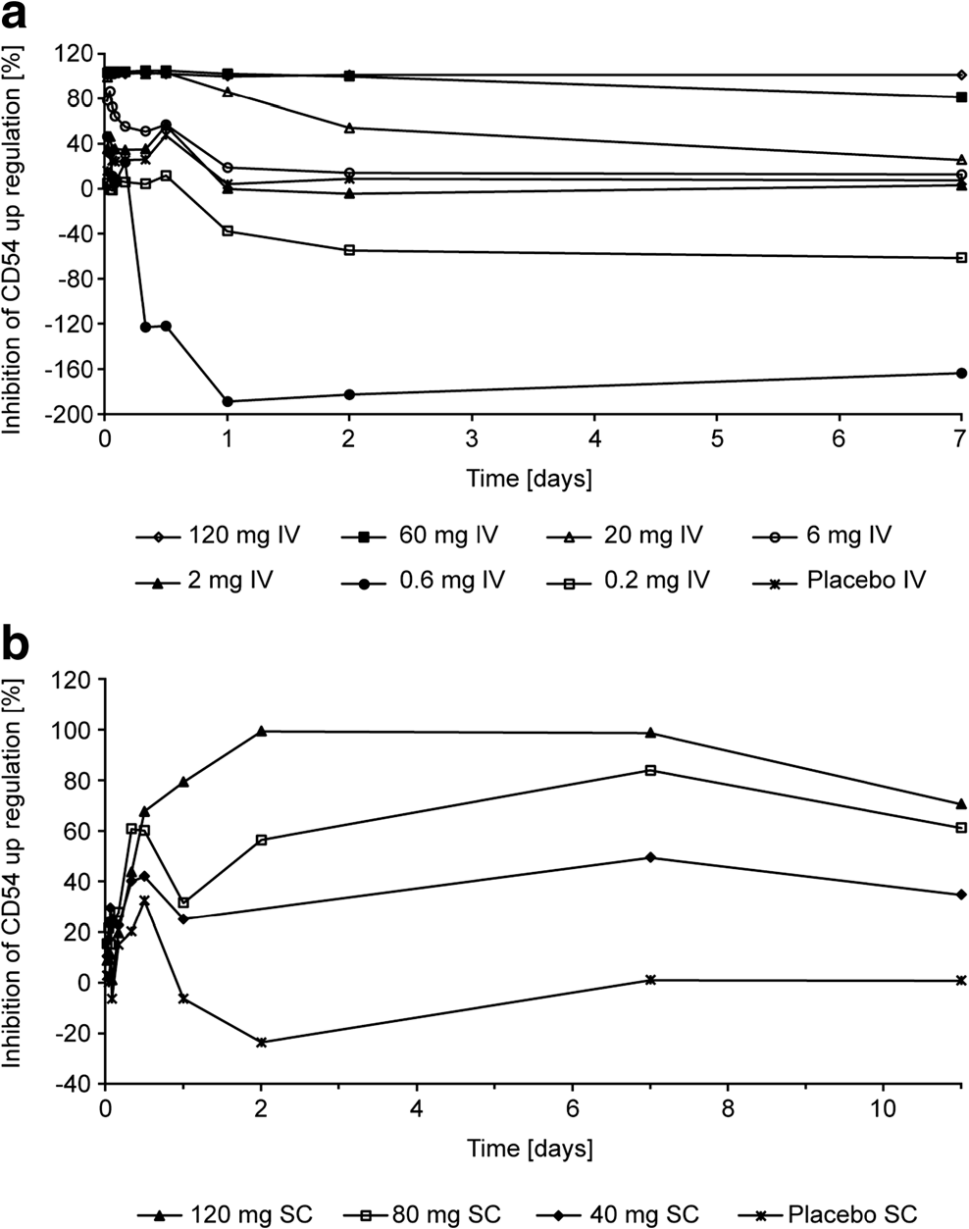
Arithmetic mean percentage of inhibition of CD54 upregulation over time after IV (**a**) or SC (**b**) administration of BI 655064. Tabulated values (mean and SD) are provided in online resource 1 Supplemental Tables S7 and S8. *IV* intravenous, *SC* subcutaneous, *SD* standard deviation

Administration of BI 655064 resulted in dose-dependent CD40 receptor occupancy and inhibition of CD54 upregulation (Figs. 2 and 3). For the IV formulation, these effects were observed at doses above 0.6 mg. Mean CD40 receptor occupancy and inhibition of CD54 upregulation after 20 mg IV infusion were maintained above 90% for 12 h after treatment. The duration of PD effect values above 90% increased to 48 h after 60 mg and at least 7 days after 120 mg in both assays. For the SC formulation, effects also increased with dose and both the mean CD40 receptor occupancy and the inhibition of CD54 upregulation above 90% were achieved at a dose of 120 mg BI 655064 from 2 h to 7 days after administration.

The sigmoidal inhibitory *E*_max_ model demonstrated a direct relationship between plasma concentration of BI 655064 and inhibition of CD40 receptor occupancy with an *E*_max_ of 94.4% (coefficient of variance [CV] 1.86%) and CD54 upregulation with an *E*_max_ of 106% (CV 1.47%). The concentrations at which EC_50_ was achieved for inhibition of CD40 receptor occupancy and CD54 upregulation were 32.8 ng/ml (CV 134%) and 61.3 ng/ml (CV 8.33%), respectively.

## Discussion

In this first-in-human trial, BI 655064 showed good overall tolerability after ascending single IV or SC doses in healthy male subjects. The most common AEs were headache and nasopharyngitis. There was one treatment-related rash and one local injection site erythema after SC injection with the 40 mg BI 655064 dose. The only SAE experienced with BI 655064 was judged by the investigator to be unrelated to treatment. The proportion of subjects with AEs and the intensity of the AEs were generally similar between active and placebo treatments and there was no dose-dependent effect.

Antibodies directed against CD40 with partial or full agonistic activity require intact Fc effector function, which can cause cytokine release and complement-mediated or antibody-dependent cellular cytotoxicity [27]. Transient B cell decreases have been reported after administration of antagonistic anti-CD40 antibodies [24, 25] and were observed after repeated administration of BI 655064 (dose range 1–50 mg/kg) in cynomolgus monkeys (unpublished data). In this trial, there were no signs of cytokine release syndrome, no significant decreases of lymphocytes or B cell subsets and no signs of immunosuppression after administration of BI 655064. Treatment with monoclonal antibodies against CD40L has been associated with an increased rate of thromboembolic events in past trials [18]. Coagulation may be mediated by immune complexes consisting of soluble CD40L and anti-CD40L antibodies, co-engagement of Fc receptors on platelets or agonistic CD40 ligation [17, 20, 28]. In pre-clinical and current clinical studies, no thromboembolic potency or events have been reported with more recently developed anti-CD40L compounds in which the Fc region has been eliminated or mutated [17, 22]. Recent trials with antagonistic anti-CD40 antibodies did not suggest an increased risk of thrombosis [23, 24]. BI 655064 is directed against CD40 and has no agonistic activity or Fc-mediated effector function that is expected to decrease the thrombotic potential. Laboratory parameters of coagulation showed minor variations but no clinically significant changes in this trial, and there were no clinical signs of thrombosis or bleeding. Immunogenicity analysis revealed seroconversion or a titre increase of ADAs in about 40% of all active subjects examined for ADAs on study day 70. ADAs did not cause any clinical symptoms or lead to observable changes in PK after single doses, but plasma concentrations of BI 655064 were already low at the time of seroconversion. The clinical significance of BI 655064 ADAs remains to be investigated in multiple dosing studies.

BI 655064 exposure increase with dose was nonlinear, as indicated by the slopes of the dose proportionality plots being well above unity. In addition, with increasing doses of BI 655064, the estimated half-life increased from 4 h to 4 days from the 6 mg to the 120 mg IV dose while clearance decreased 200-fold. These characteristics are consistent with target-mediated drug disposition [29, 30] as the predominant mechanism of clearance of BI 655064 at the doses used in this study. The effect is enhanced by the wide distribution of CD40 receptors and has been seen to some degree in other trials of an antagonistic anti-CD40 antibody [23, 25] and in pre-clinical studies of BI 655064 in cynomolgus monkeys (unpublished data). After 120 mg SC injection, BI 655064 was absorbed slowly from the site of injection and showed a lower bioavailability (30%) in total plasma exposure compared with 120 mg IV administration. The half-life after SC injection was approximately 5 days. The *E*_max_ model demonstrated a direct relationship between plasma concentration of BI 655064 and inhibition of CD40 receptor occupancy and CD54 upregulation. Both CD40 receptor occupancy and inhibition of CD54 upregulation values above 90% were achieved by doses of 20 mg IV or higher and by 120 mg SC and were maintained above 90% for up to 1 week after single doses of 120 mg IV or SC BI 655064. CD54 is an intercellular adhesion molecule on endothelial and immune cells that is upregulated following various immune signals including CD40 ligation [31, 32]. The inhibition of CD54 upregulation after incubation with MegaCD40L indicates a potent blockage of the CD40–CD40L pathway by BI 655064. The combined PK and PD results suggest the possibility of continuous inhibition of agonistic CD40 ligation with weekly IV or SC administrations of BI 655064. Based on the more than proportional PK and half-life of 4–8 days for BI 655064, it is expected that multiple weekly dosing will result in some accumulation of BI 655064, which has to be explored in a multiple rising dose study. For phase 2 (or phase 3) clinical trials, the current data suggests that a loading dose may mean the steady state is reached more quickly.

In conclusion, ascending single doses of BI 655064 were generally well tolerated. There were no relevant signs of acute immune reaction, B cell depletion or thrombosis. PK increased more than proportionally with dose, presumably due to target-mediated clearance. BI 655064 showed a high potential to block the CD40–CD40L pathway. These findings support further investigation in multiple dose trials, with continued surveillance for the signs of thrombosis and the consequences of ADA development, as well as proof-of-concept studies in autoimmune diseases including RA, SLE and LN.

## Electronic supplementary material

ESM 1 (DOCX 37 kb)
