# Supplementary material for: Safety, pharmacokinetics and pharmacodynamics of single rising doses of BI 655064, an antagonistic anti-CD40 antibody in healthy subjects: a potential novel treatment for autoimmune diseases
Source: Eur J Clin Pharmacol. 2017 Nov 10;74(2):161–9. doi: 10.1007/s00228-017-2362-8 (PMC5765193; doi:10.1007/s00228-017-2362-8)
Supplement: Supplementary file 1 — (DOCX 37 kb) [file 228_2017_2362_MOESM1_ESM.docx]

**Online resource 1.**

**Supplementary Table S1.** Baseline subject demographics

|  | Placebo | | BI 655064 | | | | | | | | | |
| --- | --- | --- | --- | --- | --- | --- | --- | --- | --- | --- | --- | --- |
|  |  | | IV | | | | | | | SC | | |
|  | IV  (*n* = 12) | SC  (*n* = 6) | 0.2 mg  (*n* = 3) | 0.6 mg  (*n* = 3) | 2 mg  (*n* = 6) | 6 mg  (*n* = 6) | 20 mg  (*n* = 6) | 60 mg  (*n* = 6) | 120 mg  (*n* = 6) | 40 mg  (*n* = 6) | 80 mg  (*n* = 6) | 120 mg  (*n* = 6) |
| Age, years | 36.3 (9.7) | 41.8 (8.7) | 42.0 (6.6) | 43.3 (6.4) | 38.3 (6.9) | 34.5 (9.4) | 33.5 (9.5) | 42.8 (5.9) | 39.7 (8.8) | 43.3 (8.8) | 43.5 (3.9) | 36.5 (8.3) |
| BMI, kg/m^2^ | 25.7 (1.9) | 27.5 (2.4) | 27.7 (2.8) | 25.9 (3.1) | 25.6 (1.0) | 24.4 (2.5) | 22.9 (3.2) | 25.0 (2.5) | 25.5 (3.5) | 24.3 (3.5) | 24.9 (3.3) | 24.6 (2.5) |

Data are arithmetic mean (standard deviation)

**Supplemental Table S2.** Pharmacokinetic parameters

| Mean (SD) | C_max_ [ng/mL] | AUC_0-tz_ [ng*h/mL] | AUC_0-∞_ [ng*h/mL] | t_max_ [h] | t_1/2_ [h] | CL* [mL/min] | V_z_* [L] |
| --- | --- | --- | --- | --- | --- | --- | --- |
| 2 mg IV (*n* = 6) | 52.2  (9.34) | 33.4  (6.83) | NC | 1.00  (0) | NC | NC | NC |
| 6 mg IV (*n* = 6) | 323  (58.9) | 532  (190) | 736  (170) | 1.00  (0.007) | 4.05  (1.16) | 142  (30.4) | 49.5  (16.5) |
| 20 mg IV (*n* = 6) | 3600  (1370) | 51,800  (33,800) | 52,800  (33,700) | 1.46  (0.459) | 15.9  (3.79) | 8.77  (4.85) | 12.7  (8.82) |
| 60 mg IV (*n* = 6) | 13,100  (2,360) | 620,000  (224,000) | 624,000  (224,000) | 1.86  (0.851) | 46.6  (10.8) | 1.80  (0.671) | 7.28  (3.57) |
| 120 mg IV (*n* = 6) | 36,100  (4,670) | 3,020,000  (645,000) | 3,030,000  (646,000) | 1.54  (0.781) | 89.2  (19.7) | 0.683  (0.124) | 5.14  (0.852) |
| 40 mg SC (*n* = 5) | 153  (110) | 21,000  (12,100) | NC | 110  (36.2) | NC | NC | NC |
| 80 mg SC (*n* = 6) | 1,100  (918) | 177,000  (152,000) | 184,000  (151,000) | 112  (12.4) | 141  (35.3) | 14.2  (10.9) | 184  (153) |
| 120 mg SC (*n* = 6) | 6,480  (4,850) | 1,360,000  (1,220,000) | 1,370,000  (1,230,000) | 108  (13.1) | 172  (209) | 3.31  (2.66) | 27.3  16.3 |

*CL and V_z_ expressed as functions of bioavailability (CL/F and V_z_/F) for SC parameters

*AUC_0-∞_* area under the concentration-time curve of the analyte in plasma over the time interval from
0 to extrapolated to infinity, *AUC_0-tz_* area under the concentration-time curve of the analyte in plasma over the time interval from 0 to the last measurable time point of the dose, *CL,* total clearance of the analyte in plasma following intravascular administration, *C_max_* Maximum measured concentration of the analyte in plasma, *IV* intravenous, *NC* not calculated, *SC* subcutaneous*, t_1/2_* terminal elimination half-life of the analyte in plasma, *t_max_* time from dosing to the maximum measured concentration of the analyte in plasma or the maximum measured biomarker effect, *V_z_* apparent volume of distribution during the terminal phase after an intravascular dose.

**Supplementary Table S3.** Mean (SD) plasma concentrations (ng/mL) of IV BI 655064

| Time (hrs) | BI 655064 | | | | | | |
| --- | --- | --- | --- | --- | --- | --- | --- |
|  | 0.2 mg  (*n* = 3) | 0.6 mg  (*n* = 3) | 2 mg  (*n* = 6) | 6 mg  (*n* = 6) | 20 mg  (*n* = 6) | 60 mg  (*n* = 6) | 120 mg  (*n* = 6) |
| –0.25 | N/A | N/A | N/A | N/A | N/A | N/A | N/A |
| 0.5 | N/A | N/A | 40.8 (9.02) | 189 (33.1) | 1080 (523) | 5360 (1470) | 14200 (2420) |
| 1.0 | N/A | N/A | 52.2 (9.34) | 323 (58.9) | 3400 (1240) | 11700 (1960) | 32800 (3880) |
| 1.25 | N/A | N/A | N/A | 191 (59.1) | 3400 (1230) | 12300 (2430) | 29100 (3460) |
| 1.5 | N/A | N/A | N/A | 136 (44.6) | 3140 (1130) | 12000 (1830) | 33300 (5260) |
| 1.75 | N/A | N/A | N/A | 108 (38.0) | 2970 (888) | 11800 (2160) | 32600 (4400) |
| 2.0 | N/A | N/A | N/A | 85.4 (36.4) | 3320 (1570) | 11700 (1850) | 31300 (4500) |
| 2.5 | N/A | N/A | N/A | 69.5 (22.6) | 2940 (1590) | 11700 (1950) | 28300 (4120) |
| 3.0 | N/A | N/A | N/A | 59.0 (18.3) | 2940 (1240) | 11600 (2980) | 32300 (5770) |
| 4.0 | N/A | N/A | N/A | 48.9 (15.9) | 2570 (992) | 11400 (2580) | 30300 (3730) |
| 6.0 | N/A | N/A | N/A | 37.5 (9.38)* | 2200 (837) | 10100 (2130) | 32600 (4420) |
| 8.0 | N/A | N/A | N/A | N/A | 2090 (883) | 9140 (1830) | 29300 (3420) |
| 10.0 | N/A | N/A | N/A | N/A | 1870 (998) | 8160 (2270) | 28900 (4790) |
| 12.0 | N/A | N/A | N/A | N/A | 1610 (999) | 8110 (2000) | 25600 (4000) |
| 24.0 | N/A | N/A | N/A | N/A | 742 (679) | 5990 (1490) | 22000 (4130) |
| 30.0 | N/A | N/A | N/A | N/A | 497 (516) | 5480 (1530) | 19400 (3170) |
| 36.0 | N/A | N/A | N/A | N/A | 336 (340) | 5360 (1330) | 17000 (2810) |
| 48.0 | N/A | N/A | N/A | N/A | 164 (166) | 4830 (1780) | 16900 (3840) |
| 72.0 | N/A | N/A | N/A | N/A | N/A | 3330 (1340) | 12300 (1680) |
| 120.0 | N/A | N/A | N/A | N/A | N/A | 1390 (944)^†^ | 8920 (2240) |
| 168.0 | N/A | N/A | N/A | N/A | N/A | 642 (571) | 5950 (1060) |
| 264.0 | N/A | N/A | N/A | N/A | N/A | 112 (65.2) | 3420 (1600) |
| 432.0 | N/A | N/A | N/A | N/A | N/A | N/A | 544 (551) |
| 648.0 | N/A | N/A | N/A | N/A | N/A | N/A | 111 (64.5) |
| 984.0 | N/A | N/A | N/A | N/A | N/A | N/A | N/A |
| 1320.0 | N/A | N/A | N/A | N/A | N/A | N/A | N/A |
| 1656.0 | N/A | N/A | N/A | N/A | N/A | N/A | N/A |

Data are arithmetic mean (SD). *NA* no descriptive statistics available. *SD* standard deviation.
*n=4.
^†^n=5.

**Supplementary Table S4.** Mean (SD) plasma concentrations (ng/mL) of SC BI 655064

| Time (hrs) | BI 655064 | | |
| --- | --- | --- | --- |
|  | 40 mg  (*n* = 5) | 80 mg  (*n* = 6) | 120 mg  (*n* = 6) |
| –0.25 | N/A | N/A | N/A |
| 0.5 | N/A | N/A | N/A |
| 1.0 | N/A | N/A | N/A |
| 1.5 | N/A | N/A | N/A |
| 2.0 | N/A | N/A | N/A |
| 3.0 | N/A | N/A | N/A |
| 4.0 | N/A | N/A | N/A |
| 6.0 | N/A | N/A | N/A |
| 8.0 | N/A | N/A | N/A |
| 10.0 | N/A | N/A | 163 (189)^†^ |
| 12.0 | N/A | N/A | 369 (505)^†^ |
| 24.0 | N/A | N/A | 1130 (1340) |
| 30.0 | N/A | N/A | 2350 (2350) |
| 36.0 | N/A | 611 (649)* | 3220 (2830) |
| 48.0 | 94.0 (69.9)* | 567 (645)^†^ | 3690 (2900) |
| 72.0 | 149 (122)* | 760 (885) | 4880 (3790) |
| 96.0 | 141 (96.1) | 1020 (922) | 5770 (4140) |
| 120.0 | 130 (70.8) | 979 (774) | 6350 (4980) |
| 168.0 | 78.2 (29.4) | 655 (549) | 4830 (4450) |
| 264.0 | 46.5 (8.15) | 229 (216) | 2530 (2800) |
| 432.0 | N/A | 62.2 (28.3) | 627 (570) |
| 648.0 | N/A | 39.7 (9.31)* | 140 (95.9)* |
| 984.0 | N/A | N/A | N/A |
| 1320.0 | N/A | N/A | N/A |
| 1656.0 | N/A | N/A | N/A |

Data are arithmetic mean (SD). *NA* no descriptive statistics available. *SD* standard deviation.
*n=4.
^†^n=5.

**Supplementary Table S5.** Arithmetic mean (SD) percentage of CD40 receptor occupancy over time after IV administration

| Time (hrs) | Placebo  (n = 12) | BI 655064 | | | | | | |
| --- | --- | --- | --- | --- | --- | --- | --- | --- |
|  |  | 0.2 mg  (*n* = 3) | 0.6 mg  (*n* = 3) | 2 mg  (*n* = 6) | 6 mg  (*n* = 6) | 20 mg  (*n* = 6) | 60 mg  (*n* = 6) | 120 mg  (*n* = 6) |
| –0.25 | 0 (0) | 0 (0) | 0 (0) | 0 (0) | 0 (0) | 0 (0) | 0 (0) | 0 (0) |
| 0.5 | 2.40 (11.4) | 11.0 (12.7) | 26.6 (11.3) | 35.6 (5.91) | 65.9 (46.5) | 91.2 (1.63) | 91.5 (1.57) | 94.4 (1.51) |
| 1.0 | 6.70 (19.1) | 12.2 (7.39) | 29.4 (18.9) | 48.1 (9.52) | 76.0 (33.9) | 91.8 (0.995) | 93.0 (1.13) | 95.4 (1.17) |
| 1.5 | 1.53 (9.73) | 7.59 (8.07) | 9.76 (15.2) | 27.6 (7.60) | 66.5 (47.8) | 90.9 (1.35) | 92.6 (1.01) | 95.5 (1.01) |
| 2.0 | –1.87 (10.1) | 4.76 (21.1) | 10.7 (5.65) | 25.5 (7.85) | 57.1 (62.7) | 91.5 (0.942) | 92.8 (1.12) | 95.3 (1.09) |
| 4.0 | –5.04 (12.1) | 3.37 (16.2) | 0.649 (7.23) | 21.4 (6.00) | 53.4 (56.3) | 92.0 (2.27) | 92.0 (1.33) | 95.5 (1.23) |
| 8.0 | –6.64 (13.9) | –1.71 (19.3) | –10.9 (12.2) | 19.9 (5.85) | 45.3 (66.4) | 92.1 (2.61) | 92.6 (1.96) | 95.6 (0.625) |
| 12.0 | –10.7 (15.5) | –0.843 (16.9) | –11.1 (11.3) | 20.3 (12.2) | 36.6 (80.5) | 92.5 (1.04) | 92.9 (1.71) | 95.9 (0.597) |
| 24.0 | –5.80 (15.8) | –2.62 (14.1) | 0.711 (6.20) | 12.9 (12.0) | 18.9 (96.1) | 89.8 (4.29) | 92.6 (1.09) | 95.0 (0.884) |
| 48.0 | –2.53 (22.4) | 3.87 (32.7) | –0.107 (38.5) | 17.1 (7.08) | -12.2 (114) | 81.4 (12.1) | 92.0 (1.50) | 95.3 (0.477) |
| 168.0 | –1.39 (17.1) | 1.50 (9.96) | –15.0 (4.22) | 16.2 (10.7) | -56.9 (178) | 33.9 (18.2) | 87.1 (6.62) | 93.6 (0.825) |

*SD* standard deviation.

**Supplementary Table S6.** Arithmetic mean (SD) percentage of CD40 receptor occupancy over time after SC administration

| Time (hrs) | Placebo  (n = 6) | BI 655064 | | |
| --- | --- | --- | --- | --- |
|  |  | 40 mg  (*n* = 6) | 80 mg  (*n* = 6) | 120 mg  (*n* = 6) |
| –0.25 | 0 (0) | 0 (0) | 0 (0) | 0 (0) |
| 0.5 | 5.43 (10.1) | –3.80 (21.8) | 6.74 (7.19) | –3.53 (7.59) |
| 1.0 | 8.67 (10.1) | –0.139 (17.6) | 15.1 (14.3) | –3.18 (9.96) |
| 1.5 | 5.63 (10.9) | –4.38 (24.2) | 13.8 (15.9) | 1.73 (10.7) |
| 2.0 | 8.20 (10.3) | 0.0433 (22.4) | 11.3 (15.3) | 2.37 (14.6) |
| 4.0 | –3.53 (10.7) | –6.61 (20.0) | 3.91 (10.1) | 10.7 (26.2) |
| 8.0 | –6.70 (17.2) | 15.0 (19.3) | 15.2 (17.9) | 34.0 (38.5) |
| 12.0 | –1.24 (22.9) | 19.2 (25.1) | 24.9 (40.6) | 62.0 (36.1) |
| 24.0 | –4.49 (8.00) | 35.4 (34.6) | 43.0 (40.4) | 85.4 (12.1) |
| 48.0 | –4.38 (13.0)* | N/A | 76.0 (19.2) | 93.6 (3.31) |
| 168.0 | –10.9 (16.6)† | 69.5 (27.1)^‡^ | 89.2 (4.15) | 92.0 (2.48) |
| 264.0 | –4.65 (21.1)^†^ | 59.1 (7.92)* | 75.0 (11.6) | 87.7 (6.84) |

*SD* standard deviation.
*n=4.
^†^n=5. ^‡^n=3.

**Supplementary Table S7.** Arithmetic mean percentage of inhibition of CD54 up-regulation over time after IV administration

| Time (hrs) | Placebo  (n = 12) | BI 655064 | | | | | | |
| --- | --- | --- | --- | --- | --- | --- | --- | --- |
|  |  | 0.2 mg  (*n* = 3) | 0.6 mg  (*n* = 3) | 2 mg  (*n* = 6) | 6 mg  (*n* = 6) | 20 mg  (*n* = 6) | 60 mg  (*n* = 6) | 120 mg  (*n* = 6) |
| –0.25 | 0 (0) | 0 (0) | 0 (0) | 0 (0) | 0 (0) | 0 (0) | 0 (0) | 0 (0) |
| 0.5 | 15.9 (23.9) | 4.89 (21.8) | 46.3 (15.1) | 35.1 (17.0) | 78.1 (19.7) | 99.1 (4.00) | 103 (2.80) | 101 (0.801) |
| 1.0 | 8.78 (15.7) | –0.721 (14.2) | 31.4 (16.9) | 46.5 (12.5) | 86.3 (12.9) | 102 (1.40) | 104 (6.66) | 102 (1.17) |
| 1.5 | 24.7 (19.9) | –1.43 (11.1) | 11.2 (43.1) | 34.7 (20.9) | 72.5 (18.0) | 103 (1.34) | 103 (2.64) | 102 (1.08) |
| 2.0 | 23.8 (25.6) | 6.73 (15.3) | 4.93 (45.4) | 35.5 (21.1) | 64.4 (16.0) | 103 (1.27) | 104 (6.53) | 102 (1.30) |
| 4.0 | 25.4 (21.6) | 5.91 (20.6) | 23.5 (22.8) | 34.2 (25.7) | 55.2 (11.3) | 104 (0.780) | 104 (6.84) | 102 (0.872) |
| 8.0 | 26.0 (26.7) | 4.17 (20.7) | –123 (245) | 35.1 (17.4) | 50.8 (17.6) | 102 (2.15) | 105 (7.47) | 103 (1.10) |
| 12.0 | 47.6 (28.3) | 11.6 (12.9) | –122 (258) | 56.1 (9.43) | 56.9 (4.71) | 103 (1.57) | 105 (6.43) | 102 (0.932) |
| 24.0 | 3.98 (26.8) | –37.6 (38.8) | –189 (312) | –0.115 (8.48) | 18.8 (8.18) | 85.8 (19.9) | 102 (2.94) | 100 (0.494) |
| 48.0 | 8.82 (39.2) | –54.6 (84.0) | –183 (311) | –4.29 (11.4) | 14.1 (22.3) | 53.9 (20.4) | 100 (1.38) | 101 (1.43) |
| 168.0 | 7.44 (32.1) | –61.3 (73.8) | –164 (307) | 3.19 (7.85) | 12.7 (14.0) | 25.8 (12.8) | 81.0 (29.2) | 101 (0.562) |

*SD* standard deviation.

**Supplementary Table S8.** Arithmetic mean (SD) percentage of inhibition of CD54 up-regulation over time after SC administration

| Time (hrs) | Placebo  (n = 6) | BI 655064 | | |
| --- | --- | --- | --- | --- |
|  |  | 40 mg  (*n* = 6) | 80 mg  (*n* = 6) | 120 mg  (*n* = 6) |
| –0.25 | 0 (0) | 0 (0) | 0 (0) | 0 (0) |
| 0.5 | 2.84 (20.3) | 10.5 (3.22) | 15.3 (17.6) | 8.77 (8.22) |
| 1.0 | –0.0830 (24.1) | 17.0 (4.55) | 21.6 (14.7) | 9.06 (29.5) |
| 1.5 | 8.69 (18.5) | 29.4 (11.5) | 15.2 (19.5) | 11.0 (23.4) |
| 2.0 | –6.36 (51.9) | 25.0 (16.5) | 23.6 (19.6) | 0.955 (30.9) |
| 4.0 | 15.0 (25.2) | 22.6 (22.4) | 27.7 (13.1) | 19.7 (20.6) |
| 8.0 | 20.2 (26.8) | 40.0 (23.1) | 60.7 (14.4) | 43.7 (19.4) |
| 12.0 | 32.4 (54.5) | 42.0 (18.5) | 60.1 (41.8) | 67.7 (44.5) |
| 24.0 | –6.42 (35.0) | 25.0 (24.9) | 31.5 (33.4) | 79.3 (32.0) |
| 48.0 | –23.7 (54.6)* | N/A | 56.3 (35.6) | 99.4 (4.66) |
| 168.0 | 1.00 (42.0)^†^ | 49.4 (18.9)^‡^ | 83.9 (21.9) | 98.7 (3.85) |
| 264.0 | 0.731 (34.5)^†^ | 34.7 (5.91) | 61.2 (21.4) | 70.5 (38.8) |

*SD* standard deviation.
*n=4.
^†^n=5.
^‡^n=3.
